# Supplementary material for: Redox imbalance is related to HIV and pregnancy
Source: PLoS One. 2021 May 21;16(5):e0251619. doi: 10.1371/journal.pone.0251619 (PMC8139510; doi:10.1371/journal.pone.0251619)
Supplement: Attachment — Submitted filename: NEW - Response to Reviewers (version_02_2021).pdf [file pone.0251619.s003.pdf]

PONE-D-20-03191, Review Report

MANUSCRIPT: "Pregnancy factor versus infection factor in HIV-infection and the redox balance"

Botucatu, February 12th 2021.

PLOS ONE

Dear Editor,

Please find enclosed the revised version of the manuscript that considered all the referees' suggestions and we changed the funding information.

We also did a careful proofreading and the resulting changes are highlighted in blue.

We appreciate very much the opportunity to improve our paper with the referees' suggestions and the possibility to resubmit the new version.

Please find below the answers to reviewer's comments.

Kind regards,

Vanessa Martinez Manfio, PhD.
Department of Tropical Diseases
Botucatu Medical School (FMB), UNESP, Brazil

Review and Author Comments

Dear reviewers, thank you very much for the questions and suggestions. Your comments were entirely pertinent and greatly improved the paper. Please find below the answers and modifications determined by the points raised in your revision. All the alterations triggered your comments are highlighted in blue in the new version of manuscript.

Editor Comment:

The key concerns noted by the reviewers relate to the reporting of the methods and the interpretation of the findings. Specifically, Reviewer 4 raise concerns about the presence of comorbidities in the study population that would limit the interpretation of the relationship between HIV and pregnancy. Additionally, Reviewer 4 noted that the sample size calculation is not presented.

Reviewer #4:

I would like to congratulate the authors for the idea of the article that presents a relevant theme and of interest to the maternal and child area. However, I have doubts about the inclusion and exclusion criteria considered in the study.

1- As described by the authors, the entire study sample came from an infectology unit. Thus, it seems to me that the presence of other comorbidities, infections and / or situations (such as tobacco use) (a fact already reported by the authors) presented by some individuals were not considered at the time of screening. This becomes worrying because it is already well discussed in the literature that most existing diseases and smoking itself lead to increased oxidative stress. Thus, there is no guarantee that the results found are specific to HIV and pregnancy.

Our infectious diseases service (SAEI-DAM) specializes in HIV/AIDS, hepatitis and HTLV. None of the included women had co-infections or opportunistic diseases. Regarding other comorbidities and drugs use, only one patient had diabetes and other two were smokers, which did not influence our results. We added all these information in the results section, lines 199-202).

2- The ideal for the data to be indisputable is that the controls present the same characteristics as the cases, excluding only the object (s) studied and this needs to be well described in the study in the method and results sessions.

The authors agree with the reviewer about the need for homogeneity between the groups so that the differences found are strictly attributed to the factors "HIV infection" and "pregnancy". So, in addition to the sociodemographic table already presented, we have included the *p* values and another paragraph containing a more clearly information about it (results section, lines 177-194). We noticed that only 3 parameters were not homogeneous between the groups: age (however, we believe that the oxidative stress markers would not undergo major changes, considering that all women were very young); time of infection and time on ART (after all, most women were diagnosed with HIV in prenatal care). The heterogeneity of these two variables was included in the study's limitations (lines 369-370).

3- Additionally, as there is no description of the sample calculation, there is no guarantee that the sample studied is sufficient to consider the extrapolation of the results presented.

We understand your concern about the lack of sample size calculation, however, as previously mentioned, the inclusion was carried out according to the universe of eligible patients who were attended at our Health Service. Therefore, according to our patient flow, we made every effort to invite all pregnant women to this study, after careful analysis of the inclusion and exclusion criteria.

Although it serves about 700 HIV-individuals, the number of pregnant women is restricted (and can be seasonal), as well as their interest in participating in the study (a large number of women refuse to participate in the study due to the 3 blood collections proposed in the study design).

Perhaps an initial sample calculation could even show the need to include more participants in this study; however, it would not be feasible with our reality to increase this number. In addition, despite the small sample size, we were still able to demonstrate statistically significant differences in relation to the various markers analyzed, which allowed us to discuss the importance of considering both factors (pregnancy and HIV infection) in the inflammatory / oxidative state of these women.

Additionally, the study ended in 2019, not allowing its continuation – of new inclusions and evaluations - since it was a doctoral thesis that has already completed in the same year. Thus, unfortunately, it is not possible now to increase the sample size.
