# Supplementary material for: Redox imbalance is related to HIV and pregnancy
Source: PLoS One. 2021 May 21;16(5):e0251619. doi: 10.1371/journal.pone.0251619 (PMC8139510; doi:10.1371/journal.pone.0251619)
Supplement: Attachment — Submitted filename: NEW- Response to Reviewers (2021)mar.pdf [file pone.0251619.s004.pdf]

PONE-D-20-03191, Review Report

MANUSCRIPT: "Redox imbalance is related to HIV and pregnancy"

Botucatu, March 24th 2021.

PLOS ONE

Dear Editor,

Please find enclosed the revised version of the manuscript that considered the last referees' suggestions. The changes are highlighted in yellow.

We appreciate very much the opportunity to improve our paper with the referees' suggestions and the possibility to resubmit the new version.

Therefore, after all favorable comments for publication, we await the final decision of the editor.

Kind regards,

Vanessa Martinez Manfio, PhD.
Department of Tropical Diseases
Botucatu Medical School (FMB), UNESP, Brazil
