# Supplementary material for: Redox imbalance is related to HIV and pregnancy
Source: PLoS One. 2021 May 21;16(5):e0251619. doi: 10.1371/journal.pone.0251619 (PMC8139510; doi:10.1371/journal.pone.0251619)
Supplement: Attachment — Submitted filename: Response to Reviewers.docx [file pone.0251619.s002.docx]

***PONE-D-20-03191, Review Report***

*MANUSCRIPT: “Pregnancy factor versus infection factor in HIV-infection and the redox balance”*

Botucatu, July 31^th^ 2020.

**PLOS ONE**

Dear Editor,

Please find enclosed the revised version of the manuscript that considered all the referees’ suggestions. We also did a careful proofreading and the resulting changes are highlighted in yellow.

Additionally, with the editor-in-chief’s and reviewers’ permission, we would like to know the possibility of changing the title (without any effect on the entire manuscript) to “**Redox imbalance is related to HIV and pregnancy**”, because we felt that the original title was too confuse (*Pregnancy factor versus infection factor in HIV-infection and the redox balance)*. If this is possible, please I wait for instructions on how to proceed.

We appreciate very much the opportunity to improve our paper with the referees’ suggestions and the possibility to resubmit the new version.

Please find below the answers to reviewer’s comments.

Kind regards,

Vanessa Martinez Manfio, PhD.

Department of Tropical Diseases

Botucatu Medical School (FMB), UNESP, Brazil

***Review and Author Comments***

Dear reviewers, thank you very much for the questions and suggestions. Your comments were entirely pertinent and greatly improved the paper. Please find below the answers and modifications determined by the points raised in your revision. All the alterations triggered your comments are in are highlighted in yellow.

*Note: We suggest changing the title, according to a letter sent to the editor, for the purpose of better understanding by readers. “****Redox imbalance is related to HIV and pregnancy****”, rather than “Pregnancy factor versus infection factor in HIV-infection and the redox balance”. However, for this change, we await the approval of you and the editor.*

**Reviewer #1:**

**In the present article the authors tried evaluate pregnancy factor versus infection factor in HIV-infection and the redox balance. I have few observations and recommendations, those are as follows.**

**1- One of the major shortcoming of the manuscript is sample size to draw meaningful conclusion. The authors should include more cases and controls to substantiate their findings.**

The authors agree with the reviewer about the small sample number and that the inclusion of new patients could result in data that are more substantial. However, the inclusion was carried out according to the universe of eligible patients who were attended at our Health Service. Although it serves about 700 HIV-individuals, the number of pregnant women is restricted, as well as their interest in participating in the study. Part of this information was included in the methodology section (lines 99-103). Additionally, the study ended last year, since it was a doctoral thesis that was completed. Thus, unfortunately, it is not possible now to increase the sample size.

**2- The main parameter of oxidative stress is total antioxidant capacity (TAC); the value of this parameter is necessary in this manuscript for evaluating antioxidant status.**

The total antioxidant capacity (TAC) is an analyte frequently used to assess the antioxidant status of biological samples and can evaluate the antioxidant response against the free radicals produced in a given disease (Spectrophotometric assays for total antioxidant capacity (TAC) in dog serum: an update doi: 10.1186 / s12917-016-0792-7), therefore not being the main parameter of oxidative stress. Furthermore, considering that pregnant women take vitamin supplements and may have a different diet, TAC could overestimate the baseline protection value, as it represents both endogenous and exogenous antioxidants. The aim of this paper was to evaluate the influence of both HIV and pregnancy, in the redox system it comprises in oxidation and antioxidant products. For this, we measure the oxidation products of lipids (malondialdehyde) and proteins (carbonylation). When these analyzes present high values, it is inferred that the reactive oxygen species are being produced in large quantities causing an oxidation of biomolecules which damage tissues and organs leading to the appearance of diseases. However, the other side of the redox system was also checked in this paper, the activity of antioxidant enzymes (SOD and CAT) that has the function of neutralizing reactive species, preventing the oxidation of biomolecules. The TAC data does not reflect damage to biomolecules, so it was not necessary to measure in this paper, we believe that the parameters evaluated respond well to the proposed objective.

**3- Between groups Comparisons was presented in an ambiguous manner for example, the following comparison was made Line 213 & 221: G1 (red): HIV-positive women, G2 (blue): HIV-negative women**

The comparison made on line 213 refers to the activity of antioxidant enzymes (SOD and Catalase) between G1 (red): HIV-positive women, and G2 (blue): HIV-negative women´. The comparison made on line 221 refers to oxidation markers (malondyaldeide and carbonilation). To better clarify, the text was corrected in the paper (lines 229-232 and 238-241).

**4- The figures does not well drawn.**

We did readjustments in the figures 1 and 2, according to your suggestion.

**5- There are some mistakes in the manuscript for example: Line 231: non-pregnant pregnant women at.**

The sentence has been corrected (lines 251-252).

**Reviewer #2:**

**The paper presents a protocol investigating the possible influence of both infection and pregnant factor in redox balance, an accrual and very important topic. The manuscript format is almost according to the authors’ instruction of the journal. The reported evidences related to the topic of the article are correct treated and used. The different aspects considered in the text follow a logical order. Nevertheless, there are some aspects of the paper which are not clear and others must be changed. Taking into account the previous research presented, inclusion and exclusion criteria, ethical issues and methods used are correctly. Some grammatical and structure errors are found that must be corrected via helps of fluent lecture. Conflict of Interest and ethical issues are declared.**

**1- Page 6 line 108 Based on what this data was obtained. Clarify please. Other comorbidities could also influence on redox indexes as infection: hepatitis C virus infection, tuberculosis, etc or degenerative: hypertension, toxic habits. Did you consider during selection process?**

None of these women had co-infections or opportunistic diseases and two of them were smokers in the moments analysed, which did not influence our results. This information was inserted in paper (lines 184-185).

**2- Page 7 line 163 Some aspects need to be clarified in relation to the number of recruited patients and statistical method. Through redox indexes analytical process of validation some variability values are obtained in terms of precision and specificity. Those alpha and beta indexes are necessary to estimate the number of individual to obtain significant statistics with 95% of confidentiality. Accordingly to the indexes used in the study is required at least 25 individuals.**

The inclusion was carried out according to the universe of eligible patients who were attended at our Health Service. Although it serves about 700 HIV-individuals, the number of pregnant women is restricted, as well as their interest in participating in the study. Therefore, the study did not have a sample calculation, as all pregnant women belonging to the Service were invited to participate in the study. Part of this information was included in the methodology section (lines 99-103).
